# Heparinised saline versus normal saline in the maintenance of invasive arterial lines in intensive care

**DOI:** 10.1186/2197-425X-3-S1-A215

**Published:** 2015-10-01

**Authors:** M Everson, L Webber, C Stace, C Sajdler, D Freshwater-Turner

**Affiliations:** University Hospitals Bristol NHS Foundation Trust, Intensive Care Unit, Bristol, United Kingdom

## Intr

Maintaining the patency of arterial lines is essential for obtaining accurate physiological measurements, enabling blood sampling and minimising the time, expense and patient discomfort incurred by line replacement. HS is commonly used to maintain and prolong arterial line patency. In 2008, a National Patient Safety Agency alert advised organisations to minimise its use for this purpose following multiple clinical incidents involving mis- selection of heparin products. Use of HS is also associated with risks such as thrombocytopenia and haemorrhage [1]. Historical studies suggest that NS is equally as effective at maintaining line patency, although recent evidence has questioned this [2].

## Objectives

We aim to assess the impact of heparinised saline (HS) versus 0.9% normal saline (NS) on arterial line patency.

## Methods

We conducted a prospective analysis of the use of HS versus NS in maintaining arterial line patency. Over a four-month period in 2014, data was recorded on all arterial lines managed in the HDU and ITU of our university teaching hospital. During the first and last month, data was collected on the use of HS and then substituted for NS for the second and third months. Data concerning duration of line insertion and reason for removal was collected from our clinical information system Innovian® and analysed using SPSS.

## Results

We prospectively collected data for 451 arterial lines: 230 flushed with HS and 221 flushed with NS. We found lines flushed with NS had a shorter lifespan (3.03 days) compared to HS (3.37 days, p = 0.39). 78 lines were removed due to blockage. Significantly more lines became blocked when flushed with NS (23.5%) versus HS (11.3%, p = 0.003), with a relative risk of 1.9 (95% CI 1.25 - 2.96). Of the 78 blocked lines, 44% needed to be replaced: 10% from the HS group and 28% from the NS group (p = 0.0006). There was no significant difference in patient age, sex, length of ITU stay or ICNARC score between the NS and HS groups.

## Conclusions

Our study demonstrates a significantly important reduction in arterial line longevity when flushed with NS compared to HS. We have determined that these excess blockages have a significant clinical impact with further lines being inserted after blockage, resulting in increased risks to patients, wasted time and cost of resources. Our findings suggest that the current UK guidance favouring NS flushes should be reviewed.
